# Zika Virus Baculovirus-Expressed Virus-Like Particles Induce Neutralizing Antibodies in Mice

**DOI:** 10.1007/s12250-018-0030-5

**Published:** 2018-05-17

**Authors:** Shiyu Dai, Tao Zhang, Yanfang Zhang, Hualin Wang, Fei Deng

**Affiliations:** State Key Laboratory of Virology, Wuhan Institute of Virology, Chinese Academy of Sciences, Wuhan, 430071 China

**Keywords:** ZIKV, Baculovirus expression system, Virus-like particles (VLPs), Neutralizing antibodies

## Abstract

The newly emerged mosquito-borne Zika virus (ZIKV) strains pose a global challenge owing to its ability to cause microcephaly and neurological disorders. Several ZIKV vaccine candidates have been proposed, including inactivated and live attenuated virus vaccines, vector-based vaccines, DNA and RNA vaccines. These have been shown to be efficacious in preclinical studies in mice and nonhuman primates, but their use will potentially be a threat to immunocompromised individuals and pregnant women. Virus-like particles (VLPs) are empty particles composed merely of viral proteins, which can serve as a safe and valuable tool for clinical prevention and treatment strategies. In this study, we used a new strategy to produce ZIKV VLPs based on the baculovirus expression system and demonstrated the feasibility of their use as a vaccine candidate. The pre-membrane (prM) and envelope (E) proteins were co-expressed in insect cells and self-assembled into particles similar to ZIKV. We found that the ZIKV VLPs could be quickly and easily prepared in large quantities using this system. The VLPs were shown to have good immunogenicity in immunized mice, as they stimulated high levels of virus neutralizing antibody titers, ZIKV-specific IgG titers and potent memory T cell responses. Thus, the baculovirus-based ZIKV VLP vaccine is a safe, effective and economical vaccine candidate for use against ZIKV.

## Introduction

Zika virus (ZIKV), first discovered in 1947 (Dick *et al.* 1952), is a mosquito-borne flavivirus and has posed a great threat to public health over the past three decades (Lessler *et al.* 2016). It has been reported that ZIKV infection is associated with microcephaly and serious neurological complications, such as Guillain–Barre syndrome (Mlakar *et al.* 2016; Miner *et al.* 2016). There are no specific vaccines or drugs available; thus, the rapid development of a safe and effective vaccine is a high priority.

Zika viruses are spherical virions that package a single-stranded, positive-sense RNA genome complexed with multiple copies of the capsid protein (C), surrounded by a host-derived lipid envelope that involves two viral structural proteins: the pre-membrane/membrane (prM/M) and envelope (E) proteins (Heinz and Stiasny 2012). High-resolution structures of mature/immature ZIKV and the ectodomain of the E protein have revealed that ZIKV displays a similar structure to other known flaviviruses (Sirohi *et al.* 2016; Prasad *et al.* 2017; Dai *et al.* 2016).

During infection of host cells, the viral genome is translated in the cytoplasm of host cells as a single open reading frame that is subsequently cleaved into three structural proteins (C, prM and E) and seven non-structural proteins by viral and host proteases (Heinz and Stiasny 2012). The new virions assemble in the endoplasmic reticulum (ER) and bud as non-infectious immature particles consisting of 60 trimeric spikes of E-prM heterodimers (Zhang *et al.* 2003). They are transported through the exocytic pathway of host cell until they reach the trans-Golgi network (TGN). In the low-pH environment of TGN, the E-prM heterodimers are reorganized into E homodimers (Yu *et al.* 2008) and the cleavage site of prM is exposed for digestion by a host furin-like protease. After prM is cleaved, the virions become infectious mature particles composed of 180 copies of the E and M proteins on the envelope. Subsequently, the newly synthesized virions are transported to the cell surface for exocytosis.

The E glycoprotein is involved in receptor binding, attachment and virus fusion during cell entry, and it represents a major target for neutralizing antibodies, which play a critical role in protection against flaviviruses (Heinz and Stiasny 2012). Thus, the E protein is the primary antigen for ZIKV vaccine development. Neutralization studies of ZIKV-confirmed convalescent human serum or plasma samples indicate that the different lineages of ZIKV represent a single serotype, with little antigenic variation and high sequence homology, suggesting that antigens produced from one lineage would provide protection against all contemporary circulating strains (Dowd *et al.* 2016a). Recently, the development of ZIKV vaccines has been accelerated by the exploration of various antigen-delivery approaches, including inactivated and live attenuated virus vaccines, DNA vaccines, RNA vaccines, protein subunits and other viral vectors-based vaccines. These candidates have been shown to provide protection against ZIKV challenge in mice and nonhuman primates (Pierson and Graham 2016; Durbin 2016; Abbink *et al.* 2016; Larocca *et al.* 2016; Pardi *et al.* 2017; Shan *et al.* 2017; Dowd *et al.* 2016b), and some have entered early clinical trials. However, safety concerns may limit the licensing of these ZIKV vaccine candidates.

Virus-like particles (VLPs) are empty, multi-protein structures resembling native virions, but they are non-infectious due to a lack of viral genetic material (Rodriguez-Limas *et al.* 2013). The antigens are present in their native conformation (without involving a replicating virus). VLPs can serve as excellent platforms for the development of efficient vaccines, since they have the ability to induce strong humoral and cellular responses, allow rapid testing of multiple candidate antigen designs and are associated with a safer manufacturing process (Liu *et al.* 2016). Studies of ZIKV (Boigard *et al.* 2017; Yang *et al.* 2017; Garg *et al.* 2017) and other flaviviruses, such as tick-borne encephalitis virus (TBEV) (Allison *et al.* 1995), dengue fever virus (DENV) (Shang *et al.* 2012) and Japanese encephalitis virus (JEV) (Du *et al.* 2015), have showed that expression of the structural proteins prM and E is sufficient for the assembly and release of VLPs that are morphologically and antigenically similar to the native virions. ZIKV VLPs have been studied in mammalian cells (Boigard *et al.* 2017; Garg *et al.* 2017) and plants (Yang *et al.* 2017), and these studies showed that co-expressing the structural (CprME) and non-structural (NS2B/NS3) proteins, or displaying the ZIKV E protein domain III on VLPs based on the hepatitis B core antigen (HBcAg), stimulated immunized mice to generate high levels of virus neutralizing antibody (NAb) titers. In addition to mammalian cells and plants, baculovirus-insect cell systems are extensively utilized for VLP production due to a number of advantages, such as lower costs, large-scale cultivation capacity, post-translational modification of the recombinant proteins that is similar to mammalian cells and the rapid growth of insect cells in animal-product-free media, which prevents contamination by mammalian pathogens (Zeltins 2013).

In this study, we introduced a method to construct ZIKV VLPs based on the baculovirus expression system. Co-expression of the prM and E proteins in insect cells enabled the formation of VLPs that were similar to ZIKV virions. The VLPs represent a promising vaccine candidate due to their potential to induce immune responses in mice.

## Materials and Methods

### Cells and Viruses

Vero cells were obtained from the American Type Culture Collection (ATCC, Manassas, VA, USA), and grown in Dulbecco’s modified Eagle’s medium (DMEM) supplemented with 10% fetal bovine serum (FBS; Gibco, Grand Island, NY, USA) at 37 °C in 5% CO_2_. *Spodoptera frugiperda* Sf9 cells were cultured in Grace’s insect medium (Gibco, Grand Island, NY, USA), pH 6.0, supplemented with 10% FBS at 27 °C. The ZIKV strain SZ-WIV01 (GenBank accession no.: KU963796), which was isolated from the serum of an imported ZIKV case in China (Deng *et al.* 2016), was obtained from Microorganisms & Viruses Culture Collection Centre (MVCCC) of Wuhan Institute of Virology. The virus was propagated in Vero cells and stored at − 80 °C. Virus titers were determined using Vero cells by the microtitration method, and they were expressed as the 50% tissue culture infective dose (TCID_50_) according to the Reed-Muench method.

### Construction of Recombinant Baculovirus

**Fig. 1:**
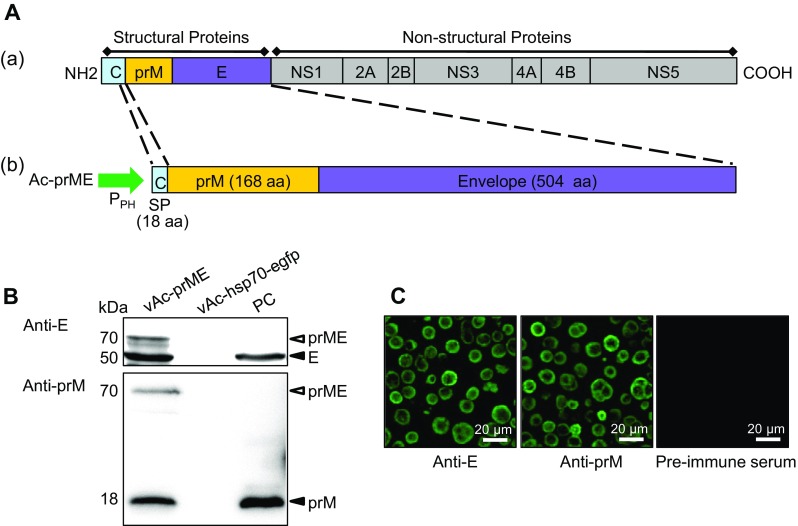
**A** Construction of recombinant baculovirus. **A**-**a** Schematic representation of the ZIKV polyprotein. **A**-**b** Structure of recombinant bacmid encoding prME for the formation of VLP in Sf9 cells. Part of prME was inserted into AcMNPV bacmid under the control of a polyhedrin promoter (P_PH_), preceded by 18 C-terminal amino acid residues of the capsid protein as the signal peptide (SP), to ensure correct expression and transportation. **B** Western blot analysis of the prM and E proteins in Sf9 cells infected with the recombinant baculovirus vAc-prME, vAc-hsp70-egfp-infected cells were used as the negative control and the ZIKV-infected Vero cells were used as the positive control. The primary antibodies used for the upper and lower panels were anti-E and anti-prM polyclonal antibody respectively. Solid triangles indicate corresponding proteins, and hollow triangles indicate the uncleaved polyprotein, prME. **C** Immunofluorescence assay of the prM and E proteins in Sf9 cells infected with the recombinant baculovirus vAc-prME. Scale bars = 20 μm.

The genomic RNA of the ZIKV strain SZ-WIV01 was extracted from infected Vero cells using TRIzol regent (Invitrogen, Carlsbad, CA, USA), and subsequently reverse transcribed using Moloney Murine Leukemia Virus (M-MLV) Reverse Transcriptase (Promega, Madison, WI, USA) according to the manufacturer’s instructions. The resultant cDNA was used as a template for the amplification of gene fragments coding for prME using the following primers: forward primer: 5′-CGGGATCCATGGGCGCAGATACTAATGTCGG-3′ and reverse primer: 5′-CGGAATTCTTAAGCAGAGACGGCTGTGGATAAG-3′. The forward primer contained a *Bam*H I restriction site and the reverse primer contained an *Eco*R I restriction site for ligation to a pFastBac™ Dual vector (Invitrogen, Carlsbad, CA, USA) under the polyhedrin promoter (P_PH_), generating pFBD-prME. To ensure the correct translocation and proper translational processing of prME, the expression cassette of prME included the signal peptide (SP) sequence derived from the C-terminus of the capsid (C) protein, as previously reported (Du *et al.* 2015; Mani *et al.* 2013) (Fig. 1A). The construct was used to generate the corresponding baculovirus, namely vAc-prME, using the Bac-to-bac system (Invitrogen, Carlsbad, CA, USA) according to the manufacturer’s instructions. The baculovirus vAc-hsp70-egfp was previously constructed in our laboratory (Wang *et al.* 2008) and was used as a control baculovirus.

### Preparation of Polyclonal Antibodies

The nucleotide sequences encoding the full-length E protein (504 aa) and prM protein (164 aa) were cloned into a pET32a vector (Novagen, Carlsbad, CA, USA). The corresponding His-tag fusion proteins were expressed in *Escherichia coli* BL21(DE3) and were purified by affinity chromatography using nickel-charged resin (Roche Diagnostics, Indianapolis, IN, USA). The purified proteins were used as antigens to generate rabbit polyclonal antiserum (anti-E and anti-prM) in our lab according to a previously reported method (Deng *et al.* 2007). Polyclonal antibodies (pAb) against GP64 and VP39 were also used (Wang *et al.* 2008).

### Western Blot Analysis of Protein Expression

The Sf9 cells were infected with the recombinant baculovirus vAc-prME or the control baculovirus vAc-hsp70-egfp at a multiplicity of infection (MOI) of 5, and the lysates were collected at 72 h post infection (h.p.i.) and prepared for sodium dodecyl sulfate polyacrylamide gel electrophoresis (SDS-PAGE), followed by transfer to polyvinylidene difluoride (PVDF) membranes (Millipore, Billerica, MA, USA). After blocking with Tris-buffered saline (TBS) containing 5% nonfat milk, the membranes were incubated with anti-E and anti-prM pAb as primary antibodies, and horseradish peroxidase (HRP)-conjugated goat anti-rabbit antibody (Sigma, St. Louis, MO, USA) as the secondary antibody. Protein band signals were detected using SuperSignal West Pico Chemiluminescent Substrate (Thermo Scientific, Rockford, IL, USA).

### Immunofluorescence

Sf9 cells were infected with the recombinant baculovirus vAc-prME at an MOI of 5. At 72 h.p.i., the cells were fixed for 10 min with 4% paraformaldehyde-PBS. The fixed cells were incubated in 0.2% Triton X-100-PBS for permeabilization, and they were then blocked with 5% bovine serum albumin (BSA; Biosharp, Hefei, China). The cells were then treated with primary antibodies for 1 h at room temperature and stained with goat anti-rabbit IgG-fluorescein isothiocyanate (FITC; Abcam, Cambridge, UK) for 1 h at room temperature. For visualization of the nuclei, the cells were incubated with Hoechst 33,258 (Beyotime, Shanghai, China) for 3 min at room temperature.

### Production and Purification of ZIKV Virions and ZIKV VLPs

Vero cells were infected with ZIKV at an MOI of 0.1. The medium was collected 4 days post infection (d.p.i.), inactivated with β-propiolactone (1:4000 v/v), cleared of cell debris and concentrated using tangential flow filtration. The sample was then loaded onto a discontinuous sucrose gradient (20%, 50%) and subjected to ultracentrifugation at 150,000 ×*g* (SW41 rotor; Beckman, Fullerton, CA, USA) for 3 h. The band between the surface of the 20% and 50% sucrose was then extracted and concentrated at 150,000 ×*g* (SW41 rotor; Beckman) for 3 h.

ZIKV VLPs were produced by Sf9 cells infected with the recombinant baculovirus vAc-prME at an MOI of 5. At 3 d.p.i., 100 mL cells (2 × 10^6^ cells/mL) were harvested by centrifugation at 3000 ×*g* for 5 min and resuspended in 10 mL cell lysis buffer (NaCl-Tris-Ethylenediaminetetraacetic acid [NTE] buffer, comprising 120 mmol/L NaCl, 10 mmol/L Tris–HCl and 1 mmol/L ethylenediaminetetraacetic acid [EDTA], pH 7.5), followed by sonication for 1 min and centrifugation at 13,000 ×*g* for 30 min. The supernatant was passed through a 0.22-μm filter to remove the debris and then concentrated using a 20% sucrose cushion at 150,000 ×*g* (SW41 rotor; Beckman) for 3 h. The pellets were resuspended in NTE buffer, sonicated for 30 s and subjected to a continuous sucrose gradient (10%–60%). After ultracentrifugation at 150,000 ×*g* (SW41 rotor; Beckman) for 3 h, 12 fractions were taken (from top to bottom) for western blot analysis and the E and prM antigen-enriched fractions were pelleted again at 150,000 ×*g* (SW41 rotor; Beckman) for 3 h. The pellets were resuspended in 100 μL NTE buffer for subsequent transmission electron microscopy (TEM) assays.

### TEM and Immune-Electron Microscopy (IEM)

To observe VLPs within cells, Sf9 cells were infected with vAc-prME at an MOI of 5. Infected cells were harvested at 72 h.p.i. and processed for electron microscopy as previously described (Vanlent *et al.* 1990) with slight modification. Sf9 cells infected with vAc-hsp70-egfp and healthy cells were used as controls.

After purification, the VLPs or ZIKV particles were adsorbed onto formvar-coated copper grids for 5 min, negatively stained using 2% phosphotungstic acid (PTA) for 1 min and then examined with a transmission electron microscope (H-7000 FA; Hitachi, Japan).

For IEM, purified particles were adhered onto carbon-coated nickel grids (200 mesh) and blocked with 5% BSA. The primary antibodies were anti-prM and anti-E pAb and rabbit pre-immune serum. The 12-nm Colloidal Gold-AffiniPure Goat Anti-Rabbit IgG (Jackson ImmunoResearch, West Grove, PA, USA) was used as the secondary antibody. Subsequently, the grids were negatively stained and examined with TEM as described above.

### Mice Immunizations

Four groups of 6–8-week-old female BALB/c mice (n = 6 in each group) were vaccinated with 50 μg ZIKV VLPs, 5 μg purified inactivated ZIKV vaccine (designated PIV, for positive control), 50 μg vAc-hsp70-egfp-infected Sf9 cell lysates (designated NC, for negative control; these cells were treated under exactly the same conditions as the ZIKV VLPs) or PBS (blank control). The level of E and prM proteins in purified inactivated ZIKV and VLPs used in a dose were analyzed by Western blot analysis and quantified by ImageJ software. All vaccines were adjuvanted with aluminum hydroxide (Thermo Fisher, Waltham, MA, USA) using a 1:1 volume ratio, and they were administered via the intramuscular (i.m.) route at weeks 0, 2 and 4. Serum samples were taken before and 2 weeks after each vaccination to monitor the humoral immune response. All the animals were euthanized, and the splenocytes were harvested at week 6 for cellular immune response studies. The animal experiment procedures were approved by the ethics committees of Wuhan Institute of Virology, Chinese Academy of Sciences (approval number: WIVA33201601).

### Microneutralization Assay

The neutralization test was carried out using a microneutralization assay in Vero cells. The sera were incubated at 56 °C for 30 min to inactivate the complement. Vero cells in 96-well plates were cultured overnight to 80% confluence. For each well, 50 μL of a serial two-fold dilution of the serum was mixed with 50 μL (equal volume) of 100 TCID50 of ZIKV and incubated at 37 °C for 1 h to neutralize the infectious viruses. The mixtures were then transferred to the Vero cell monolayers. After incubation for 5 days at 37 °C, the NAb titer, defined as the highest dilution of serum that resulted in a 50% reduction in the cytopathic effect, was recorded.

### Serum Enzyme-Linked Immunosorbent Assay (ELISA)

ZIKV-specific antibodies (IgG, IgG1, IgG2a, IgG2b, IgG2C, IgG3 and IgM) in sera were determined by ELISA. Microplates (Xiamen Labware, Xiamen, China) were coated with 50 μL/well of purified and inactivated ZIKV (2 μg/mL) or VLPs (5 μg/mL) at 4 °C overnight. After extensive plate washing and blocking with 5% (w/v) BSA in PBS with Tween 20 (PBST) for 1 h at 37 °C, the serum was serially diluted and added to the wells in triplicate. Following 1 h incubation at 37 °C, the plates were washed and HRP-conjugated goat anti-mouse secondary antibodies (Jackson Immuno Research, West Grove, PA, USA) were added. After 1 h of incubation at 37 °C, the plates were washed and developed by adding 100 μL/well of 3,3′,5,5′-tetramethylbenzidine (TMB) substrate in the dark at room temperature for 20 min and stopped with 50 μL of 2 mol/L H_2_SO_4_. The optical density at 450 nm (OD450) of the plates was read using an ELISA plate reader (BioTek, USA). The titer for each group was determined as the reciprocal of the highest serum dilution with OD value 2σ above the mean of the negative control.

### Cytokine Detection

Memory immune response was measured at 2 weeks after the last immunization using a Mouse IFN-γ Precoated ELISPOT kit (Dakewe Bioengineering, Beijing, China) according to the manufacturer’s instructions. Briefly, 3 × 10^5^ splenocytes/well (in duplicate) were cultured with inactivated ZIKV (5 μg/mL) or ZIKV VLPs (10 μg/mL) as an antigenic stimulator. Phorbol 12-myristate 13-acetate (PMA; 50 ng/mL) with ionomycin (1 μg/mL) was used as the positive control. Spots were counted using a Bio-Reader (ByoSys, German).

To detect cytokine production, 2 × 10^6^ splenocytes/well were cultured in 24-well plates with 0.5 mL Roswell Park Memorial Institute (RPMI) 1640 medium (containing 10% FBS) and stimulated with ZIKV (5 μg/mL) or ZIKV VLPs (10 μg/mL). After incubation at 37 °C for 48 h, the media were collected, softly centrifuged at 1000 rpm for 5 min and assayed for interferon (IFN)-γ, interleukin (IL)-2, IL-4 and IL-10 production using commercially available ELISA kits (4A Biotech, Beijing, China). All the assays were performed in triplicate.

### Statistics Analysis

For the statistical analysis of antibody titers, the titers were first transformed to log10.

Data are shown as the mean ± standard deviations (SD) of six mice per group. Statistical significance was determined by Student’s *t* test, with *P* value < 0.05 considered to be statistically significant.

## Results

### Recombinant Baculovirus Expressing ZIKV Proteins

The DNA fragment encoding ZIKV prME was inserted into AcMNPV bacmid under the control of the polyhedrin promoter, which generated the recombinant bacmid Ac-prME (Fig. 1A). After transfection and infection, the recombinant baculovirus, vAc-prME, was generated and confirmed using western blot and immunofluorescence assays (IFA) (Fig. 1) using specific antibodies. As shown in Fig. 1B, separate bands corresponding to prM (18 kDa) and E (50 kDa) proteins were detected, indicating that digestion processing performed by host cell signalase had indeed occurred at the native cleavage site. In addition, a 70-kDa band corresponding to the uncleaved polyprotein prME was also detected. In the ZIKV infected Vero cells, which were used as positive control, the prME band was not detected. This could because the cleavage of prME in ZIKV-sensitive Vero cells is more efficient and complete.

### ZIKV VLPs Were Generated by the Baculovirus Expression System

**Fig. 2:**
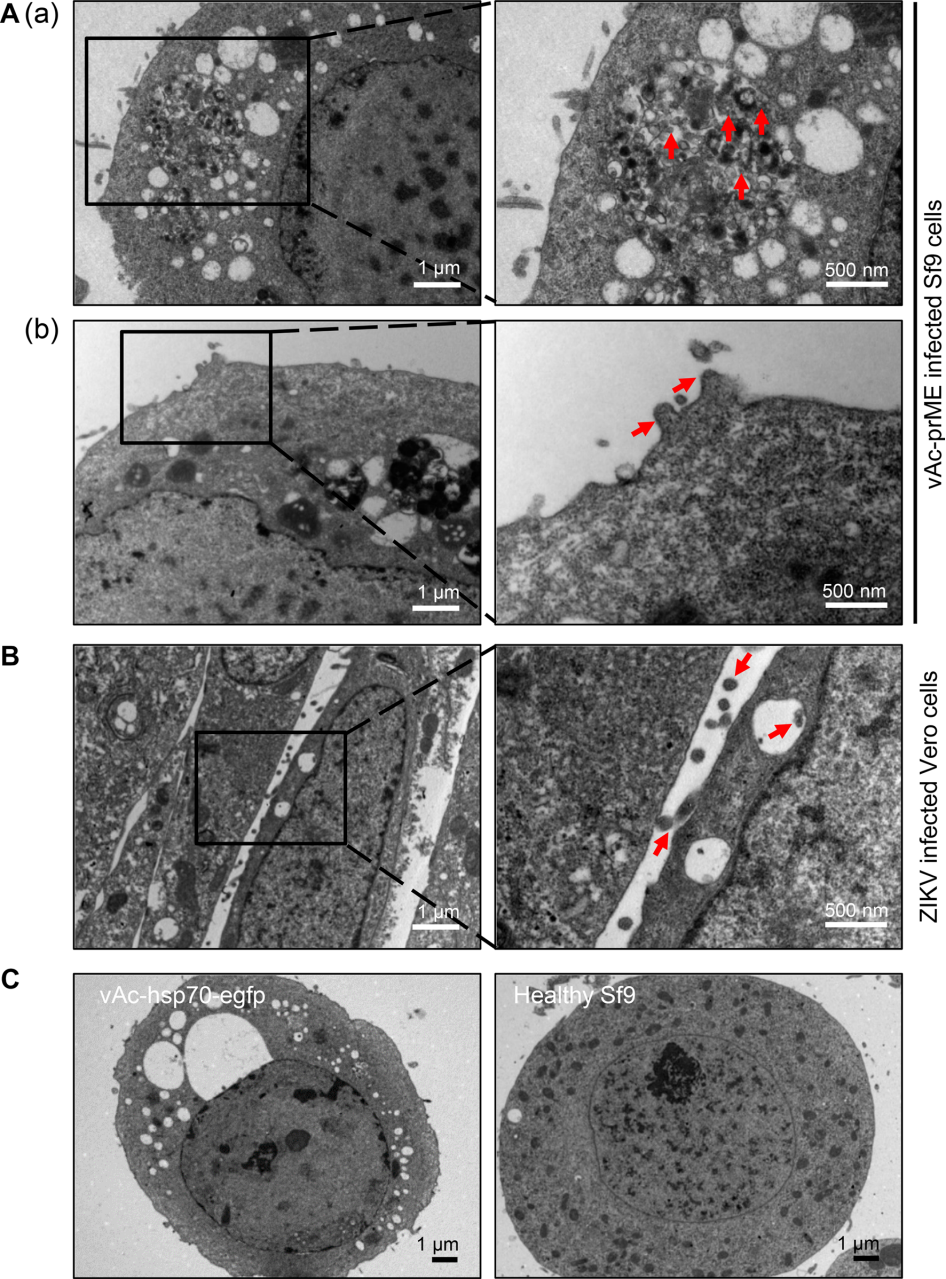
Ultrathin sections of vAc-prME-infected Sf9 cells and ZIKV-infected Vero cells. **A** Transmission electron micrographs of Sf9 cells infected with vAc-prME. **A**–**a** Vesicles filled with small spherical particles (identified by the arrows) in Sf9 cells infected with vAc-prME. **A**–**b** Spherical particles presumed to be ZIKV VLPs budding from the cell membrane. **B** Transmission electron micrographs of Vero cells infected with ZIKV. The virions bud from the cell membrane. **C** Transmission electron micrographs of Sf9 cells infected with vAc-hsp70-egfp and healthy Sf9 cells. For **A** and **B**, the right panels show enlarged views of the boxed regions in the corresponding left panels.

The Sf9 cells infected with the recombinant baculovirus vAc-prME were harvested at 72 h.p.i. and observed using TEM. As indicated in Fig. 2A, Sf9 cells infected with vAc-prME had vesicles full of spherical particles ranging from 50 to 100 nm in diameter, which were presumed to be VLPs, and some particles were released from the cell surface, similar to the native ZIKV exocytosis process from Vero cells (Fig. 2B). In contrast, Sf9 cells infected with vAc-hsp70-egfp and healthy Sf9 cells had empty vesicles or no vesicle (Fig. 2C).

**Fig. 3:**
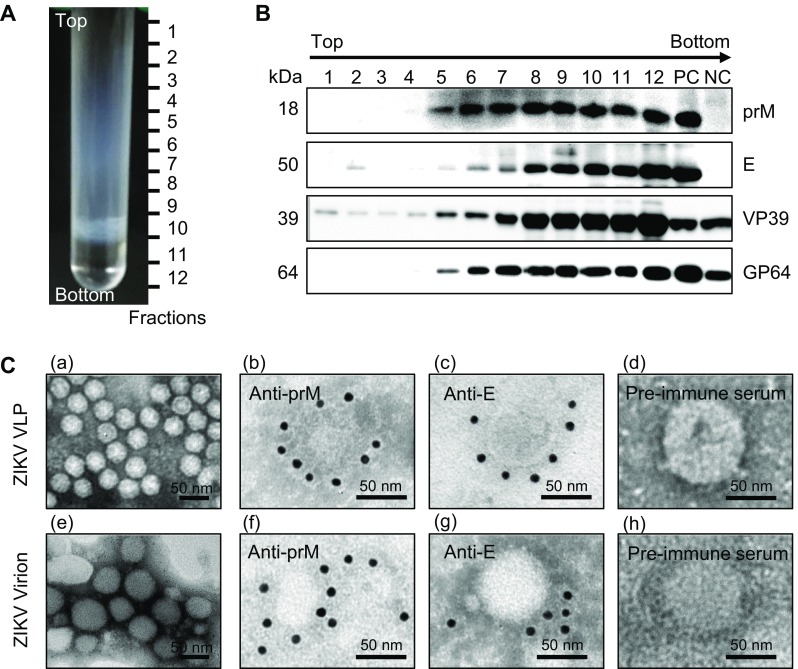
Characterization of baculovirus-expressed ZIKV VLPs. **A** Lysates of vAc-prME-infected Sf9 cells were layered onto 10%–60% sucrose gradients and subjected to centrifugation. Twelve fractions were taken from top to bottom. **B** Western blot analysis of purified sucrose gradient fractions using the indicated antibodies. **C** Electron micrographs of negative staining and immunogold labeling of VLPs and ZIKV. **C**–**a** Negative staining of purified ZIKV VLPs from the E and prM antigen-enriched fractions from the sucrose gradient; IEM of VLPs using anti-prM **C**–**b**, anti-E, **C**–**c** polyclonal antibodies and rabbit pre-immune serum, **C**–**d** as primary antibodies; **C**–**e** Negative staining of purified ZIKV; IEM of ZIKV using anti-prM **C**–**f**, anti-E, **C**–**g** polyclonal antibodies and rabbit pre-immune serum, **C**–**h** as primary antibodies; Scale bars = 50 nm.

Lysates of vAc-prME-infected Sf9 cells were layered onto the 10%–60% sucrose gradients and subjected to ultracentrifugation. After ultracentrifugation, 12 fractions were taken (from top to bottom) for western blot analysis. As shown in Fig. 3A, 3B, ZIKV-specific antigens were distributed across the sucrose gradient, from 30% to 60%. Electron microscopic analysis of negative-stained purified ZIKV-antigen-rich fractions from the vAc-prME-infected Sf9 cell lysates revealed rough spherical particles of 30-50 nm in size, which resembled native ZIKV virions in morphology (Fig. 3C). IEM using anti-E/anti-prM antibodies further revealed immunogold particles surrounding distinct particles, which were similar to native virions (Fig. 3C), indicating the exposure of E and prM on the outer surface of the VLPs. All these data demonstrate that ZIKV VLPs comprising prM and E proteins can be assembled in Sf9 cells infected with vAc-prME and purified from the lysates by sucrose gradient ultracentrifugation.

### ZIKV VLPs Elicited Neutralizing Antibodies and Virus-Specific IgG

**Fig. 4:**
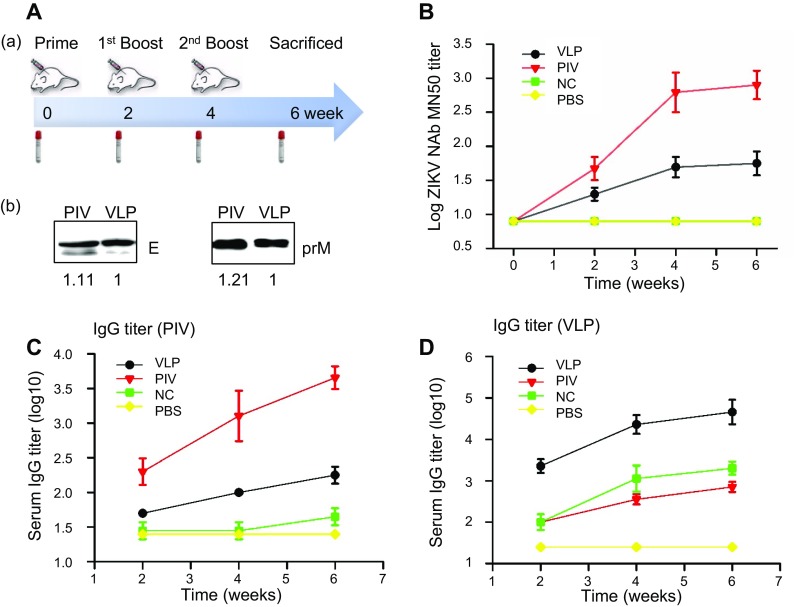
Antibody responses elicited by ZIKV VLPs or control vaccinations. **A A**–**a** BALB/c mice (n = 6) were immunized with three doses of ZIKV VLPs (VLP), purified inactivated ZIKV vaccine (PIV), vAc-hsp70-egfp-infected Sf9 cell lysates (negative control, NC) or PBS at weeks 0, 2 and 4. Blood samples were collected at weeks 0 (pre-immune serum), 2, 4 and 6 (2 weeks after each antigen injection). **A**–**b** The purified inactivated ZIKV and VLPs used in a dose (5 μg PIV and 50 μg ZIKV VLPs) were analyzed by Western blot analysis and quantified by densitometry, the level of E (left panel) and prM (right panel) proteins in PIV relative to VLP were 1.11: 1 and 1.21: 1. **B** Neutralization titers of the immunized mice determined by a microneutralization assay. **C** ZIKV-specific IgG titers and **D** VLP-specific IgG titers were measured via ELISA using two coating antigens: purified inactivated ZIKV or ZIKV VLPs. The values represent the mean ± SD of the reciprocal of the highest serial dilution (with an OD450 value 2σ above the mean of the negative control) of six mice per group. The results of three independent experiments (with technical triplicates for each sample) are presented.

BALB/c mice were immunized via the i.m. route three times at 2-week intervals with VLPs or the control immunizations (*i.e.*, the positive control involving purified inactivated ZIKV, the negative control involving vAc-hsp70-egfp-infected Sf9 cell lysates or the blank control involving PBS). Based on the gray intensity of the bands in the Western blot analysis, the doses of E and prM proteins in PIV relative to VLP used to immunize mice were relevant as ratio of 1.11: 1 and 1.21: 1 respectively (Fig. 4A). Serum samples were collected at 2 weeks after each immunization, and evaluated for humoral immune responses induced by VLPs and control vaccinations.

To demonstrate whether the antibodies induced by VLPs were able to neutralize live ZIKV, a microneutralization assay was carried out and the geometric mean titer (GMT) was calculated for each group (Fig. 4B). In VLP-immunized mice, neutralization titers after primary immunization were significantly increased compared with the pre-immune serum (week 0). Antibody titers at week 6 (2 weeks after the second booster) were similar to those at week 4 (2 weeks after the first booster), suggesting that the last immunization did not significantly further boost the neutralization response. The neutralization titers elicited by VLPs were lower than those elicited by inactivated ZIKV, but significantly different from those elicited in the other two control groups.

**Fig. 5:**
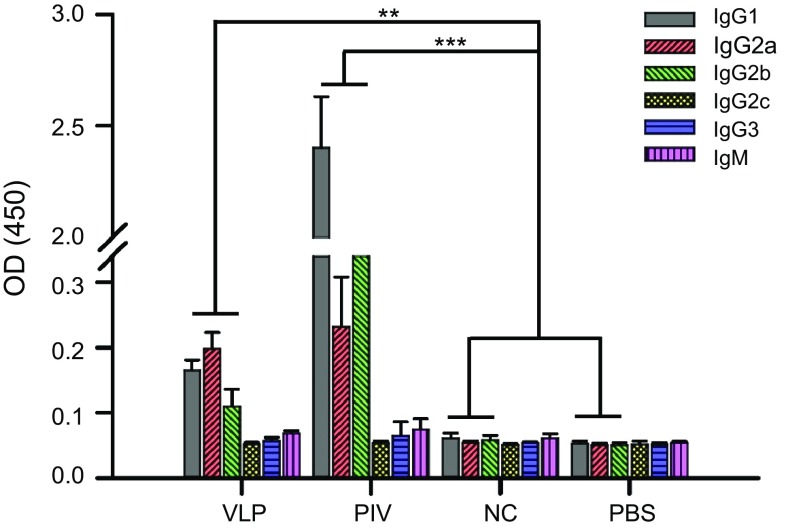
Subclasses of antibody responses elicited by ZIKV VLPs or control vaccinations. ZIKV-specific subclass antibodies (IgG1, IgG2a, IgG2b, IgG2c, IgG3 and IgM) in all immunized mice were measured using ELISA. The sera were diluted at 1:400. The values represent the mean ± SD of six mice per group. The results of three independent experiments (with technical triplicates for each sample) are presented. *P < 0.05, **P < 0.01; ***P < 0.001.

**Fig. 6:**
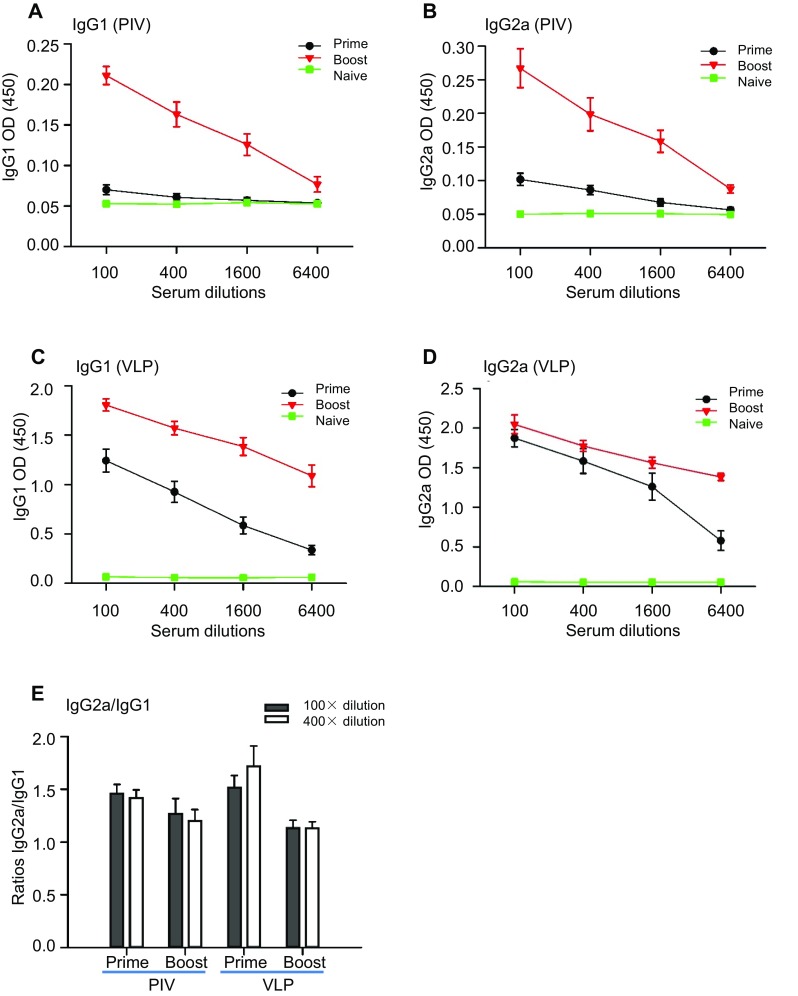
Th1/Th2 IgG isotypes elicited by ZIKV VLPs. **A** ZIKV-specific IgG1 antibodies in ZIKV VLP-immunized mice. **B** ZIKV-specific IgG2a antibodies in ZIKV VLP-immunized mice. **C** VLP-specific IgG1 antibodies in ZIKV VLP-immunized mice. **D** VLP-specific IgG2a antibodies in ZIKV VLP-immunized mice. **E** Ratios of IgG2a to IgG1 antibodies in ZIKV VLP-immunized mice. The values represent the mean ± SD of six mice. The results of three independent experiments (with technical triplicates for each sample) are presented.

As shown in Fig. 4C, 4D, when purified VLPs were used as the coating antigen, mice immunized with ZIKV VLPs had a significantly higher level of specific serum IgG compared with the control mice. In addition, both inactivated ZIKV (positive control) and vAc-hsp70-egfp-infected Sf9 cell lysates (negative control) induced higher IgG responses than PBS (blank control). In account of the assembly process of VLPs, except for the specific ZIKV antigens, there should have some other proteins were packaged into or mixed with the VLPs, like the host cells proteins or baculovirus proteins, which may strengthen the immunogenicity of the VLPs. However, VLPs induced lower ZIKV-specific IgG titers than inactivated ZIKV when we used inactivated ZIKV as the coating antigen, yet, after immunization, the level was dramatically increased compared with the level in the other two control groups. Specific IgG titers were also lower when the plates were coated with inactivated ZIKV than VLPs. Since the ZIKV VLPs were co-purified with some process-related contaminants, which shown adjuvant activity and induced antibodies that were not specific to ZIKV. In the serum from mice immunized with ZIKV VLPs, antibodies could recognize the VLP antigens finely, but only the ZIKV-specific antibodies could react with the inactivated ZIKV antigens and led to low ZIKV-specific IgG titers. To further investigate the immune profiles induced, we analyzed the antibody subclasses in all immunized animals. Mice immunized with ZIKV VLPs or inactivated ZIKV induced evident IgG1, IgG2a and IgG2b antibodies specific for ZIKV, but IgG2c, IgG3 and IgM antibodies were not detected at substantial levels (Fig. 5). The profile of IgG2a/IgG1 was considered to be an indicator of the polarization of T helper (Th)1 and Th2 cell-mediated immune responses. After primary immunization with VLPs, the IgG2a/IgG1 isotype ratio was approximately 1.5, suggesting that VLP immunization resulted in mostly the induction of Th1 cell-mediated immune responses. The two booster VLP immunizations increased the levels of both IgG1 and IgG2a, resulting in a decrease in the IgG2a/IgG1 ratio, indicating that the Th2 cell-mediated responses were also increased (Fig. 6). Overall, these results show that the VLPs were able to generate broadly ZIKV-specific antibodies capable of neutralization.

### ZIKV VLPs Induced Virus-Specific T cell Responses

**Fig. 7:**
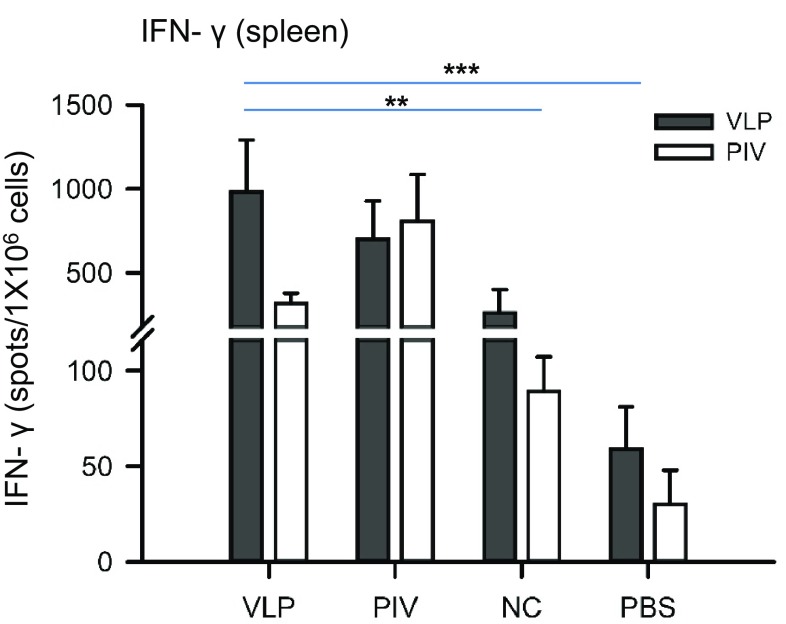
IFN-γ-secreting memory splenocytes in immunized mice. All the mice were euthanized at week 6 (2 weeks after the last immunization), and the spleens were collected and assayed. Splenocytes were isolated and stimulated with 10 μg purified ZIKV VLPs (VLP) or 5 μg purified inactive ZIKV (PIV). IFN-γ-secreting cells were identified by ELISpots after stimulation. All data are presented as the mean ± SD of six mice in each group. The results of two independent experiments (with technical duplicates for each sample) are presented. *P < 0.05, **P < 0.01; ***P < 0.001.

The responses of Th cells play an important role in generating both humoral and cellular immune responses. Th1-cells are known to be involved in cell-mediated immunity, while Th2-cells function as helper T-cells in humoral immunity (Ch’ng *et al.* 2011). To determine the ability of ZIKV VLPs to induce specific anti-ZIKV T cell immune responses, an enzyme-linked immunospot (ELISpot) assay for detecting IFN-γ-secreting cells was performed using splenocytes of immunized mice that were collected 2 weeks after the last immunization. As depicted in Fig. 7, splenocytes from mice immunized with ZIKV VLPs and inactivated ZIKV showed significantly higher levels of IFN-γ-secreting cell spots (in response to VLPs or inactivated ZIKV antigens) compared with both control groups (the vAc-hsp70-egfp and PBS groups). In addition, the number of IFN-γ-secreting cells detected among the splenocytes stimulated with VLPs was significantly higher than the number of spots detected among those stimulated with inactivated ZIKV for all groups of immunized mice apart from the inactivated ZIKV group (the positive control), as expected.

**Fig. 8:**
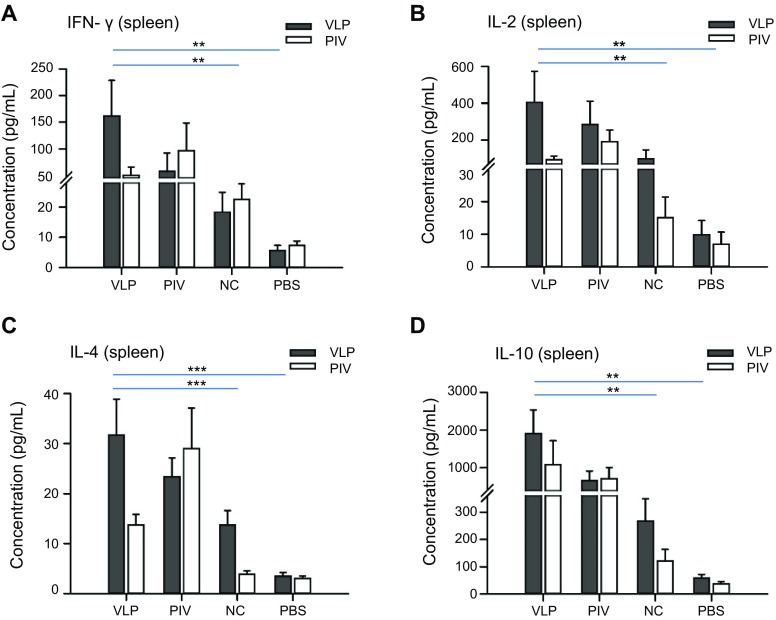
Proliferation and cytokine production of splenocytes from immunized mice. Splenocytes were isolated and stimulated with 10 μg purified ZIKV VLPs (VLP) or 5 μg purified inactive ZIKV (PIV). The supernatants were then collected after 48 h of incubation and used to measure the concentrations of **A** IFN-γ, **B** IL-2, **C** IL-4 and **D** IL-10 using ELISA. All data are presented as the mean ± SD of six mice in each group. The results of three independent experiments (with technical triplicates for each sample) are presented. *P < 0.05, **P < 0.01; ***P < 0.001.

The production of cytokines by splenocytes was measured as indicators of the memory T cell response (Del Prete and Romagnani 1994). The Th1 (IFN-γ and IL-2) and Th2 (IL-4 and IL-10) cytokines produced by splenocytes stimulated with VLPs or inactivated ZIKV were measured by ELISA. The results showed that splenocytes from the mice immunized with VLPs and inactivated ZIKV generated substantially higher levels of IFN-γ and IL-2 than the levels in the other two control groups, indicating the induction of Th1 cell-mediated immune responses (Fig. 8A, 8B). The levels of IL-4 and IL-10 also indicated similar results (Fig. 8C, 8D), though the detection of IL-4 is generally difficult owing to its low expression levels. Th2 cell-mediated immune responses were also evoked. Furthermore, we found that in response to ZIKV antigens, the cytokines in inactivated ZIKV-immunized mice were higher than in VLP-immunized mice except for IL-10. All these results suggest that VLPs are able to induce memory T cell responses like inactivated ZIKV.

## Discussion

Recently, several types of ZIKV vaccine candidates, including inactivated and live attenuated viruses, DNA and RNA vaccines, have been shown to have protective efficacy (Durbin and Wilder-Smith 2017). While these vaccine candidates show great promise, difficulties remain to be overcome regarding their licensing, especially on account of safety. The use of live viruses increases the risk of viral dissemination, and these vaccines increase the risk of side effects due to infection with viral nucleic acids. As alternative ZIKV vaccine platform, ZIKV VLPs have been produced in mammalian cells and plants and have been demonstrated to be potentially safe and effective (Boigard *et al.* 2017; Yang *et al.* 2017). VLPs are highly ordered multiprotein structures and carry many characteristics of viruses that can be used in vaccine development. Their good safety profile makes ZIKV VLPs an appropriate choice for immunocompromised individuals and pregnant women, as the World Health Organization has identified women of child-bearing age (including pregnant women) as the primary target population for ZIKV vaccination. Furthermore, VLPs have high immunogenicity and efficacy due to their unique structures. Flavivirus infection usually elicits neutralizing antibodies that bind only to complex quaternary epitopes that are only displayed on intact particles, and not to recombinant monomeric E proteins (de Alwis *et al.* 2012). ZIKV VLPs display E proteins with proper folding and conformation, which means that they have advantages over subunit vaccines (such as the single E protein vaccine) regarding the induction of neutralizing antibodies.

In this study, we described a new strategy for generating ZIKV VLPs consisting of prM and E proteins in insect Sf9 cells using recombinant baculovirus, and these VLPs can potentially induce strong humoral and cellular immune responses in mice. The VLPs were efficiently isolated using sucrose gradient purification. TEM and IEM revealed that the VLPs appear as rough spherical particles and have similar morphology and antigenicity to native virions. However, the lack of an inner shell of capsid protein and the viral genome leads to a smaller size (30–50 nm) compared with native virions.

An animal experiment was performed to determine the immunogenicity and efficacy of this VLP-based vaccine, and a summary of the results is shown in Table 1. The serum samples from the mice before and after immunization were assayed. The ZIKV-specific IgG and NAb levels obviously increased after immunization with VLPs. However, VLPs seemed to result in lower NAb titers than inactivated ZIKV. This may be partially due to the conformation of VLPs potentially being slightly different compared with the conformation of the authentic virus, as this conformational change may affect the neutralizing capacity and binding capacity to ZIKV. We also speculated that the ZIKV-specific antigens in our VLP vaccine were not as much as those in the inactivated virus vaccine and different vaccination dosage should be administered in further experiment. Moreover, an antibody subclass switch occurred, as demonstrated by the findings regarding the ZIKV-specific IgG subclasses in the serum. The ELISA results showed that in VLP-immunized mice, more IgG2a than IgG1 was initially produced. However, after two booster injections, the levels of both IgG2a and IgG1 isotypes increased and were not significantly different, suggesting that booster immunizations led to memory B-cell activation and promote the Th1 immune response switch to a more balanced Th1/Th2 immune response. Immunization with VLPs also elicited robust memory T cell immune responses, which are important for viral clearance (Del Prete and Romagnani 1994). The production of high levels of Th1 type cytokines (IFN-γ and IL-2) as well as Th2 type cytokines (IL-4 and IL-10) indicates that a mixed Th1/Th2 response was primed successfully by VLPs. All these results indicate that the VLP-based vaccine is highly immunogenic (inducing a wide-ranging and balanced immune response), however, the neutralization antibody titers and cytokine levels were relatively weak compared with those elicited by the inactivated virus. This may be due to the lack of proper protein processing and post translational modifications in the baculovirus-insect system, and the proper folding, especially glycosylation of viral antigens plays an important role in the efficacy of VLP-based vaccines as it is critical for immune recognition (Rudd *et al.* 1999). However, lepidopteran cells are unable to produce glycoproteins with structurally authentic mammalian N-glycans (Jarvis 2003). Although the immune responses elicited by the VLPs are significantly weaker than those elicited by inactivated ZIKV, VLPs certainly represent a safer method of preventing ZIKV infection compared with other clinical prevention strategies. In addition, the VLP production process involving insect cells is low risk and fast. The protective immunity of the VLPs *in vivo* and the potential of the VLPs to induce antibody-dependent enhancement of infection (ADE) will be investigated further in a suitable animal model, e.g., A129 (type I interferon receptor-deficient) or wild-type mice treated with an anti-IFNAR1 antibody.

**Table 1:** Immune responses elicited by the VLP vaccine and controls

	VLP	PIV	NC	PBS
Dose (μg)	50	5	50	–
NAb (log10)	1.8	2.9	0.9	0.9
IgG (log10)	4.7/2.3	2.9/3.7	3.3/1.6	1.4/1.4
IFN-γ (spots)	928/318	702/803	263/89	59/30
IFN-γ (pg/mL)	161.4/50.0	58.2/97.0	18.4/22.6	5.7/7.3
IL-2 (pg/mL)	403.9/93.5	285.2/191.7	98.8/15.1	9.8/7.0
IL-4 (pg/mL)	31.7/13.7	23.4/29.0	13.7/3.9	3.5/3.1
IL-10 (pg/mL)	1915.1/1085.5	668.7/714.0	268.2/121.7	59.2/36.9

VLP, ZIKV VLP vaccine; PIV, purified inactivated vaccine; NC, negative control (vAc-hsp70-egfp-infected Sf9 cell lysates); PBS, blank control. NAb and IgG show the geometric mean titers of neutralization and virus-specific titers in serum collected at week 6. For IgG titers, the left values represent the VLP-specific IgG titers and the right values represent ZIKV-specific IgG titers. IFN-γ (spots) indicates the number of IFN-γ-secreting splenocytes per 1 × 10^6^ cells. For IFN-γ, IL-2, IL4 and IL-10, the left and right values represent the production stimulated by VLPs and inactivated ZIKV, respectively. n = 6 for all groups.

We propose that ZIKV VLPs produced by insect cells using recombinant baculovirus should be further developed as a safe and effective vaccine candidate to protect humans against ZIKV outbreaks.
